# Hardening Mechanism of Coral Aggregates in Cement-Based Systems

**DOI:** 10.3390/ma16010214

**Published:** 2022-12-26

**Authors:** Huajin Wang, Minglei Shi, Xintao Tian, Yue Zhang, Jinyu Liu

**Affiliations:** School of Transportation, Southeast University, Nanjing 211189, China

**Keywords:** coral concrete material, single coral particle, crushing strength, particle fragmentation test, Weibull distribution, interfacial transition zone, nanoindentation test

## Abstract

In terms of the infrastructure construction near coral reefs, native coral aggregates have been widely implemented as alternative efficient building materials to prepare the “coral concrete”. This study focused on the mechanical properties and hardening mechanism of coral particles under cement-based systems. Firstly, coral particles were divided into two categories: coral biological debris particles (calcium sand) and coral parent rock particles (limestone). Subsequently, several elementary laboratory tests were conducted to compare the physical and chemical properties of the samples. The results demonstrate that the specific surface area and open pores of coral particles are bigger than those of quartz sand. Moreover, the water absorption rate of debris and parent rock particles reach 9.9% and 22%, respectively. To further examine the hardening mechanism of coral particles, we carried out particle crushing strength tests, compressive strength tests and nanoindentation tests. Regardless of the tested groups and particle categories, the results show that the wrapped cement slurry universally demonstrated the successful enhancement of crushing strength *σ*_0,*d.*_ Particularly, in the size range from 1.18–2.36 mm, wrapped particles of debris and parent rock both reached unusually high *σ*_0,*d*_ values: 10.14 MPa and 8.74 MPa, respectively. However, in the size range from 9.5 mm to 16 mm, the *σ*_0,*d*_ values of wrapped debris and parent rock reached 4.75 MPa and 3.18 MPa, respectively. According to the nanoindentation tests, the sub-microscopic strength of ITZs was enhanced to more than 1 GPa, which is higher than that of conventional concrete, in terms of the sample with 28-day maintenance. In conclusion, this study has provided a further basis for studying coral concrete material and its hardening mechanism.

## 1. Introduction

Over recent years, governments around the world unanimously have accorded abiding significance to the construction of offshore island infrastructure. Correspondingly, there has been an important research topic about which alternative efficient building materials can be implemented to replace traditional cement aggregates [1,2]. Fortunately, one native material, i.e., coral aggregate, can provide a relatively excellent approach. Therefore, such natural advantages have drawn a growing amount of attention from several scholars on manufacturing coral concrete to replace traditional cement concrete [3,4,5].

However, the distinction in physical properties between coral particles and land-derived sand radically contributes to their differentiated mechanical properties [6]. The natural marine deposition process generally makes the layers of coral reef unfragmented and unmilled, which preserves the original morphological character of biological remains and parent rock fragments, molding the coral particles with richly angular and irregular shapes, and rough on the surface [7].

Research on coral parent rock particles has shown that these particles are primarily destroyed by tension, with the majority of these destructions occurring along the reef limestone growth line [8]. The parent rock also has a large structure, which results in seriously reduced mechanical properties after disturbance [9]. The coral parent rock is classified as soft rock, with hard and brittle characteristics [10]. In terms of the study of calcareous sand, Wang et al. [11] through scanning electron microscope (SEM) analysis revealed that there are a lot of pores on the surface of particles, and particles of a size between 0.5–2.0 mm are more irregular. Jiang et al. [12] analyzed the apparent porosity of calcareous sand with different particle sizes and kinds. The porosity is observed to decrease with increasing particle size and is found to be more affected by particle shape when the particle size is bigger than 1 mm. According to the microstructure investigation, calcareous sand has a high angle and irregularly shaped particle characteristics. Additionally, the particles have numerous internal pores [13]. Furthermore, coral particle shape and porosity have a major impact on their mechanical characteristics [14]. Wu and Ma [15,16] investigated the effect of particle size and shape on the crushing strength of coral particles and discovered that the crushing strength decreased as particle size increased. Additionally, as the calcium carbonate content increased, the particle crushing strength decreased [17,18].

As was mentioned above, studies have demonstrated that coral particles have heterogeneous geometry, a rough surface, a high specific surface area and abundant open pores on the surface. To fill the pores in coral concrete, a high cement content is necessary [19]. The high water absorption rate of coral particles will have an impact on the water–cement ratio, which will then have an impact on the overall strength of the coral concrete [20,21]. In addition, the coral particles are soft, have low hardness, and have weak crushing strength. The coral coarse aggregates with soft and low hardness characteristics cannot serve as skeletal supports for coral concrete. The coral fine particles with irregular particle shapes affect the overall fluidity [15,22,23]. Because of the particularity of coral materials, it is necessary to study the hardening characteristics of coral particles under the cement-based system.

In conclusion, these earlier studies mostly focused on the intrinsic characteristics of coral particles, while few gave enough weight to the hardening mechanism of coral particles in cement-based systems. To fill in the gaps of coral particles research, this study first investigated the specific gravity, water absorption rate, specific surface area and open pores of coral particles, to analyze the specificity of different coral particles. Additionally, through the particle crushing experiments, we analyzed the hardening mechanism of coral particles under different particle sizes before and after wrapping cement slurry. To examine the hardening mechanism of coral particles in the concrete system, coral concrete also undergoes compressive strength tests and nanoindentation tests. Consequently, this work serves as a foundation for examining the mechanical formation mechanism and macroscopic mechanical properties of coral concrete.

## 2. Materials and Methods

### 2.1. Materials

The coral particles used in this study were obtained with cutter suction dredgers from the South China Sea. According to JGJ 52-2006 [24], the coral particles were screened with a sieve shaker, and the sizes varied from 0.075 mm to 19 mm. The particle size distribution can be seen in Table 1. Additionally, the part basic characteristics of the coral particles were measured according to JGJ 52-2006 [24]. The bulk density of the coral particles was 733 kg/m^3^, while their apparent density was 1636 kg/m^3^, their porosity was 55.2%, and their water absorption ranged from 10.1% to 21.9%.

**Figure 1:**
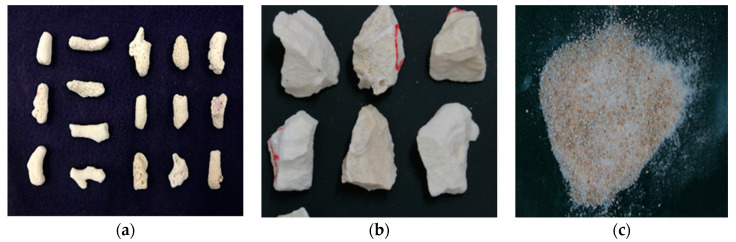
Typical particles: (**a**) coral biological debris particles; (**b**) coral parent rock particles; (**c**) ordinary quartz sand.

Furthermore, in this study, the coral particles were separated into coral biological debris (calcium sand) and coral parent rock (limestone). On the one hand, these two fundamentally significant compositions display a similar chemical mechanism as a result of the same carbonatite-induced sedimentation and diagenesis. On the other hand, due to the continuous combined effect of sedimentary facies, the diagenesis environment and tectonic processing, a long-term transformation mechanism between coral debris and coral reef rocks existed. Considering the practical application in engineering, the subsequent laboratory experiments focused on the coral parent rock particles (0.6–16 mm) and coral biological debris particles (0–16 mm) in Figure 1.

Considering the short period of construction and the large volume of engineering tasks required for island construction, this paper considered the use of early-strength cement. The cement used in the tests was P.II 42.5R silicate cement produced by Nanjing Jiangnan-Onoda Company, and the basic properties of cement are shown in Table 2.

The concrete admixture used in this study was Sika ViscoCrete 540P modified polycarboxylate high-efficiency water-reducing agent. The basic properties are listed in Table 3.

### 2.2. Method

#### 2.2.1. Specific Surface Area and Pore Volume

The specific surface area and pore volume were measured following GB/T 19587-2017 [25]. Before testing, all samples were dried and cooled to room temperature. The samples were then placed in sample tubes and degassed under vacuum at 100~350 °C. Subsequently, they were moved to the analytical station for nitrogen adsorption experiments. The instrument ASAP 2020V3.00H (Micromeritics Instrument Corporation, Norcross, GA, USA) was employed for measuring the specific surface area and pore volume [26].

#### 2.2.2. Specific Gravity

The specific gravity of coral particles was measured according to GB/T50123-2019 [27]. There are three common methods for determining specific gravity: the specific gravity bottle method (d < 5 mm), the suspending weigh method (d ≥ 5 mm, no more than 10% particles d ≥ 20 mm) and the siphon-can method (d ≥ 5 mm, more than 10% particles d ≥ 20 mm).

For the specific gravity bottle method, use a 100 mL pycnometer bottle filled with 15 g of dry sand with particle sizes less than 5 mm. Boil the sand to remove the air before using. Subsequently, place pure water into the pycnometer, wait for the bottle suspension stability, wipe off the outside water, weigh the combined weight of the bottle, water, and sand, and measure the temperature of the water in the bottle. The specific gravity is calculated according to Equation (1).

(1) $$G_{s}=\frac{m_{d}}{m_{bw}+m_{d}-m_{bws}}G_{wt}$$

where $m_{d}$ is the quality of dry sand. $m_{bw}$ means the quality of specific gravity bottles and water. $m_{bws}$ represents the quality of specific gravity bottles, water and dry sand. $G_{wt}$ is the specific gravity of water at *T* °C.

For the suspending weigh method, weigh 1 kg of the samples, wash them, put them in water for 24 h, take them out and wipe the surface water. After that, place them in the wire basket, submerge them in water, weigh the combined weight of the wire basket and samples in the water, and measure the temperature of the water. Calculate the specific gravity with Equation (2).

(2) $$G_{s}=\frac{m_{d}}{m_{d}-(m_{ks}-m_{k})}G_{wt}$$

where $m_{d}$ is the quality of specimens. $m_{ks}$ means the quality of samples and wire-basket in the water. $m_{k}$ represents the quality of the wire basket in the water.

#### 2.2.3. Water Absorption Rate

**Figure 2:**
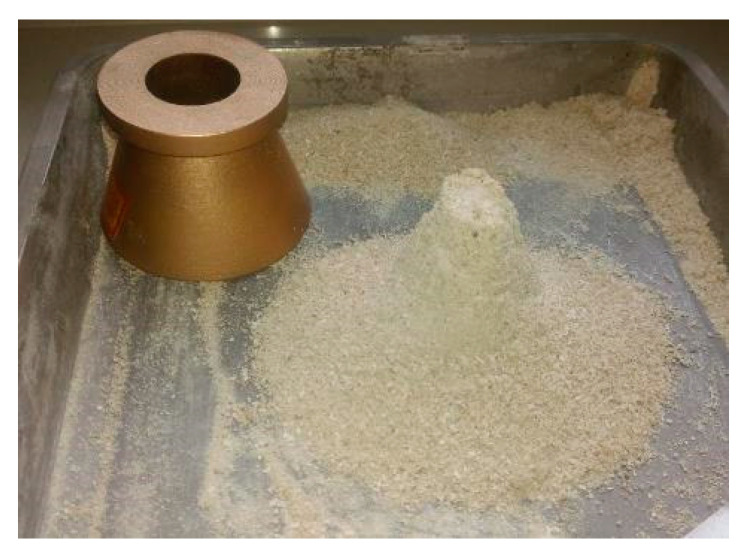
The saturated surface dry test mould.

The water absorption rate of coral particles was tested according to JGJ 52-2006 [24]. For the fine particles, the water absorption rate was tested by saturated surface dry test mould, which can be seen in Figure 2. The first step is to weigh 500 g of saturated surface dry samples and put them into a beaker with mass m_1_. After that, dry them to constant weight in an oven at 105 °C, and then cool them to room temperature in the dryer. Weigh the combined weight of dry samples and beaker which is recorded as m_2_, and calculate the water absorption according to Equation (3).

(3) $$\omega =\frac{500-(m_{2}-m_{1})}{m_{2}-m_{1}}\times 100\%$$

The gravel water absorption test method was used for the coarse particles. After soaking the samples in water for 24 h, dry the surface water with a wet towel, put them into a shallow pan and weigh them. The weight of samples and the shallow pan is recorded as m_2_. Place the saturated surface dry sample and the shallow pan in an oven at 105 °C and dry it to constant weight. After cooling, weigh the combined weight of the dried samples and shallow pan, which is recorded as m_1_, and weigh the mass of the shallow pan, which is recorded as m_3_. Calculate the water absorption rate according to Equation (4).

(4) $$\omega =\frac{m_{2}-m_{1}}{m_{1}-m_{3}}\times 100\%$$

#### 2.2.4. Diffraction of X-rays (XRD)

For XRD analysis, the coral particles were washed and dried to constant weight in an oven at 60 °C. Before the test, the coral particles were ground and passed through the 0.075 mm size sieve. The D8-Discover X-ray diffractometer (Brooke, Karlsruhe, Germany) was used for the test, and the step width was set as 0.02°, the scan speed as 0.15 s/step, and the measuring angle (2θ) as 10°–90°.

#### 2.2.5. Scanning Electron Microscopy (SEM)

In this study, scanning electron microscopy (Guangzhou Jiarui Scientific Instrument Co., Ltd, Guangzhou, China) was used to observe the composition and shape of coral particles. After crushing, different particles of about 50 mm^3^ were selected for observation. The samples were sprayed with gold (Pt) before observation. We selected an appropriate angle for taking pictures.

#### 2.2.6. Crushing Strength of Single Particle

According to JGJ 52-2006 [24], coral parent rock (limestone) particles and coral biological debris (calcium sand) were separated from coral particles and subsequently divided into fine particle groups (1.18 mm ≤ d < 2.36 mm, 2.36 mm ≤ d < 4.75 mm) and coarse particle groups (4.75 mm ≤ d < 9.5 mm, 9.5 mm ≤ d < 16 mm). Subsequently, the samples of crushing strength tests were prepared with PII42.5R cement, a water–cement ratio of 1:2 and a water reducing agent (0.05%). The coral particles were mixed with the paste for 3 min and then placed at 20 °C and 95% RH for standard curing for 7 days.

**Figure 3:**
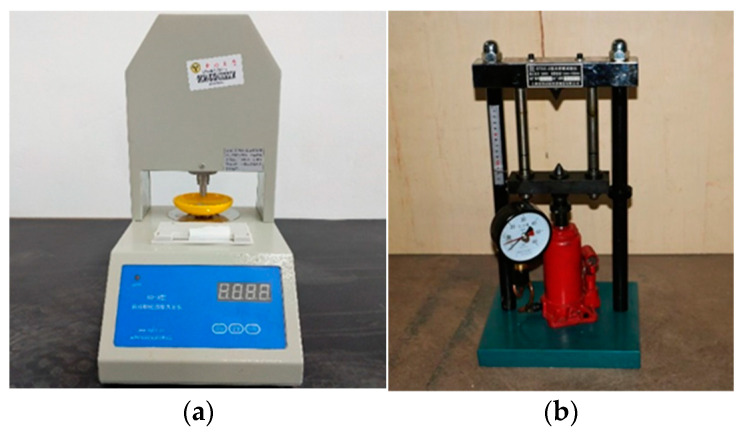
Particle crushing strength instrument: (**a**) high-pressure particle anvil device KQ-2; (**b**) point load instrument.

To assess the crushing strength of the coral particles, the high-pressure particle anvil device KQ-2 (Jiahang Bochuang Technology Co., Ltd, Beijing, China) was implemented for fine particle groups (d < 4.75 mm) and coarse particle groups (4.75 mm < d < 9.5 mm). The instrument can be seen in Figure 3a. Equation (5) was utilized to calculate the crushing strength tested by the high-pressure particle anvil device.

(5) $$\sigma_{f}=0.9\frac{F_{f}}{d^{2}}$$

where d is the average diameter of the single coral particle with cement slurry wrapped. $F_{f}$ means the critical crushing stress. $\sigma_{f}$ represents the crushing strength.

**Figure 4:**
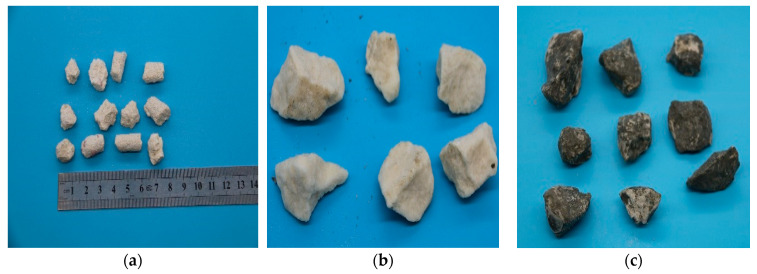
The samples of particle crushing strength: (**a**) coral biological debris particles; (**b**) coral parent rock particles; (**c**) the wrapped coral parent rock particles.

While the point load instrument (Cangzhou Lanqi Instrument Co., Ltd, Hebei, China) was applied for coarse particle groups (d ≥ 9.5 mm), Equation (6) was utilized to calculate the crushing strength tested by the point load instrument. The instrument can be seen in Figure 3b. More notably, all specimen groups were tested in single-particle form regardless of whether wrapping cement slurry was used or not. The samples can be seen in Figure 4.

(6) $$\sigma_{f}=10\pi \frac{F_{f}}{4DW_{f}}$$

where D is the spacing of loading points. $F_{f}$ means the critical stress when the crushing occurred. $W_{f}$ refers to the width of the failure surface. $\sigma_{f}$ represents the crushing strength.

#### 2.2.7. Nanoindentation Test

**Figure 5:**
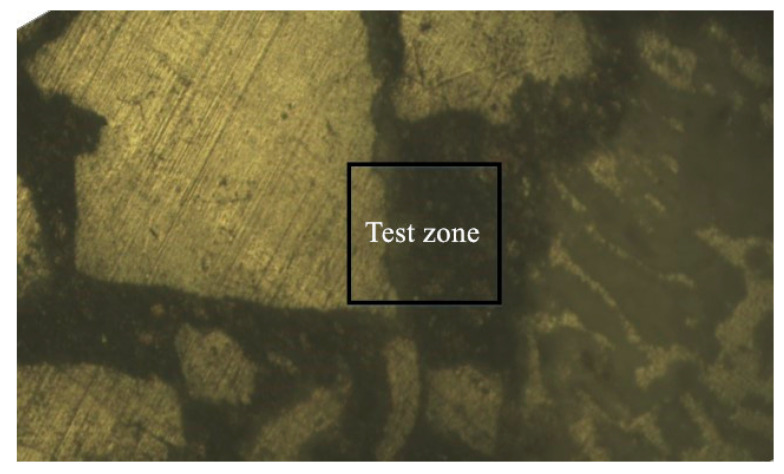
The test zone of the nanoindentation test.

The samples prepared for the nanoindentation tests were collected from the coral concrete after standard curing for 3 days and 28 days. The samples were then sliced, milled and produced as 40 mm × 40 mm × 10 mm cubes. The samples were selected with coral coarse aggregates and hardened cement paste. The test zone of sample can be seen in Figure 5.

## 3. Results and Discussion

### 3.1. The Basic Characteristics of Coral Particles

The specific surface area and open pore volume of coral particles were compiled in Table 4. Exactly speaking, the specific surface area of coral biological debris more than tripled in those of coral parent rock particles and ordinary quartz sand. Similarly, the open pore volume of the former one was over four times greater than that of the latter two. These results corresponded to the special characteristics of the particles of coral biological debris, such as extremely irregular shape, distinct angularity and rough texture. It is noteworthy that the open pore volume of coral parent rock particles was still significantly higher than that of ordinary quartz sand; in other words, both coral particles commonly demonstrate larger specific surface area and open pore volume. Last but not least, both debris and parent rock particles demonstrated a distinct inverse correlation of specific surface area and pore volume to particle size ranges.

In general, specific gravity serves as a key index for assessing porosity ratio and compressibility. It is defined as the ratio of particle mass to pure water mass under the same volume. Specific gravity is generally determined by the mineral composition of the granular material. For instance, the specific gravity of quartz sand is generally 2.65.

As shown in Table 5, coral particles were classified into fine particle group S (d < 4.75 mm) and coarse particle group G (d ≥ 4.75 mm), and the two groups were measured with the specific gravity bottle method and the siphon-can method, respectively. The results revealed that the specific gravity of coral particles ranged from 2 to 3 regardless of fine or coarse particle groups. However, the ranges of specific gravity were dependent on the categories of coral particles, specifically coral biological debris ranging from 2.5 to 3 and coral parent rock fragments from 2 to 2.5. It is greatly meaningful to compare these values with those of ordinary quartz sand at 2.65 when applied in practical engineering.

The studied particles for the water absorption rate ranged in size from 2.36 mm to 16 mm. According to Table 6, coral parent rock particles presented an average water absorption of up to 22.02%, twice that of coral biological debris particles at 9.90%. It is a reasonable explanation for such significant disparity that the former particles were characterized with abundant surface open pores and excellent internal connection between those pores. Conversely, the latter particles displayed more abundant surface open pores while worse internal connection between those pores. The last but not least, regardless of tested groups, the varied particle size had few influences on water absorption rates.

### 3.2. Micro Analysis

**Figure 6:**
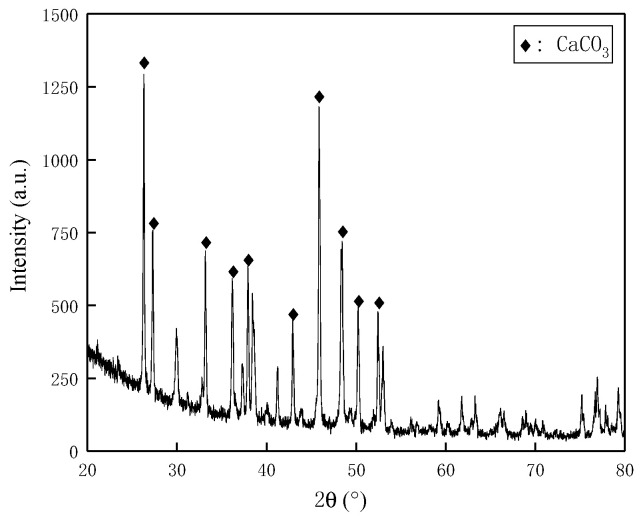
X-Ray diffraction pattern of coral particles.

In this study, the X-ray diffraction method was implemented to determine the mineral constituent of coral particles, and the diffraction pattern can be seen in Figure 6. As is presented in Table 7, the X-ray fluorescence spectrometric analysis was correspondingly applied to identify the mass proportion of various chemical compounds in coral particles. These results collaboratively revealed the mineral constituents as majorly aragonite, high-magnesian calcite, lesser dolomite, ankerite and a few quartzes. Therefore, the essential chemical compound was calcium carbonate and its equivalent carbonate content reached beyond 97%. Moreover, the Moh’s hardness of these main carbonate minerals (commonly ranging from 3 to 4) was significantly less than that of quartz (around 7), which indicated the relatively lower crushing strength level of coral particles compared to that of ordinary quartz sand.

In general, coral particles present three significant geometric characteristics, specifically irregular particle shapes, rough surfaces and large open pores. As a native aggregate, it plays a vital role in the strength performance and workability of coral concrete. Therefore, it is of great importance to conduct further research on the geometric characteristics of coral particles for practical engineering applications.

**Figure 7:**
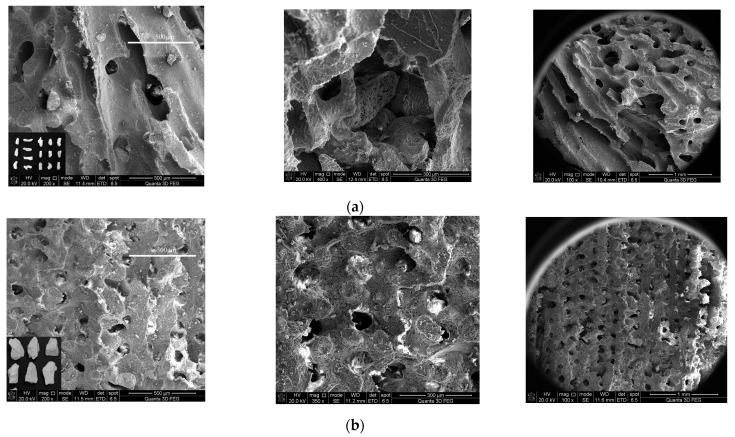
SEM images of coral particles: (**a**) coral biological debris particles; (**b**) coral parent rock particles.

To intuitively compare the surface structure of coral particles, this study chose the magnification of 100, 200 and 400 times to observe the structure of different coral particles. The SEM images can be seen in Figure 7. The coral debris particles have more open pores on their surface and better connectivity with the interior. Additionally, the shape of the particle is more irregular and the specific surface area is bigger. Due to geological sedimentation, the open pores in coral parent rock particles are relatively tiny and neatly arranged. Furthermore, the parent rock particles have more regular open pores, but the inner connectedness is inferior.

### 3.3. Hardening Mechanism of Single Coral Particle

Since several research groups [15,28] have tested the crushing strength of coral particles collected all over the world, the coral particle is commonly defined with high brittleness due to low hardness and worse shear strength. To offset such typically adverse characteristics, it is a common practice to use the wrapped cement slurry in practical engineering for hardening coral particles. Furthermore, Weibull statistical analysis was used in this section to investigate the hardening mechanism of the single coral particle in cement-based systems.

#### 3.3.1. Theoretical Basis of Weibull Statistical Analysis

The proposal of Weibull Statistical Theory is fundamentally important to describe the exponential distribution of experimental data, particularly for reliability study, lifetime assessment, quality control monitoring and material tests [29]. Owing to its wide applicability, it has been utilized in various fields with significant achievements. For instance, as a typical extreme value normal distribution, Weibull Statistical Theory has generally been applied to characterize the strength level and range of brittle (or quasi-brittle) materials in practical engineering, such as concrete [30], asphalt mixtures [31] and coral sand particles [15]. Similarly, this study has implemented the Weibull empirical correlations as the following Equations (7)–(9) to calculate survival probability $P_{s}$ and the normalized effective crushing strength of a single coral particle $\sigma_{m}$.

(7) $$P_{s}=\exp\left[-V{\left(\frac{\sigma -\sigma_{u}}{\sigma_{0}}\right)}^{m}\right]$$

where V is the specimen unit volume (often as 1). $P_{s}$ means the survival probability and m means the Weibull modulus. σ represents the measurement of particle crushing strength, $\sigma_{0}$ represents a scale parameter with the same dimensions as σ and $\sigma_{u}$ represents a boundary stress value when the survival probability $P_{s}=1$; in other words, $\sigma_{u}$ is a cut-off stress below which the particles never fail.

(8) $$\sigma_{m}=\int_{\sigma_{u}}^{\infty }P_{s}d\sigma =\sigma_{0,d}\Gamma \left(1+\frac{1}{m}\right)$$

where $\sigma_{0,\;d}$ represents the characteristic crushing strength when the particle diameter is d and survival probability $P_{s}=37\%$, and $\sigma_{m}$ represents the normalized effective crushing strength of a coral single particle after Weibull statistical analysis.

(9) $$P_{s}=\frac{n-i}{n}$$

where n is the total number of tested specimens. i means the rank of one tested single particle among all specimens.

#### 3.3.2. Crushing Strength of Coarse and Fine Single Particle

**Figure 8:**
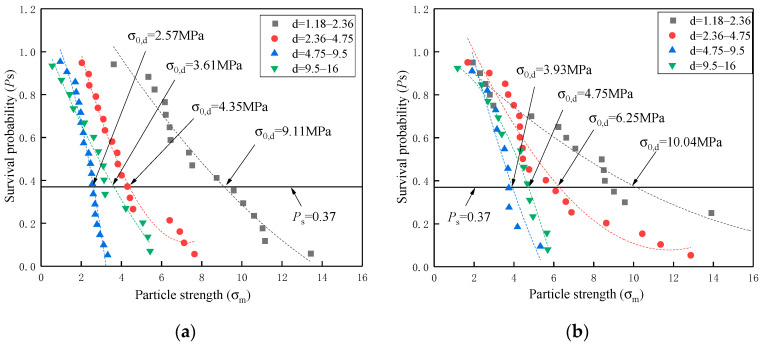
The curves of survival probability to crushing strength of coral biological debris particles: (**a**) without wrapping cement slurry; (**b**) with wrapping cement slurry.

**Figure 9:**
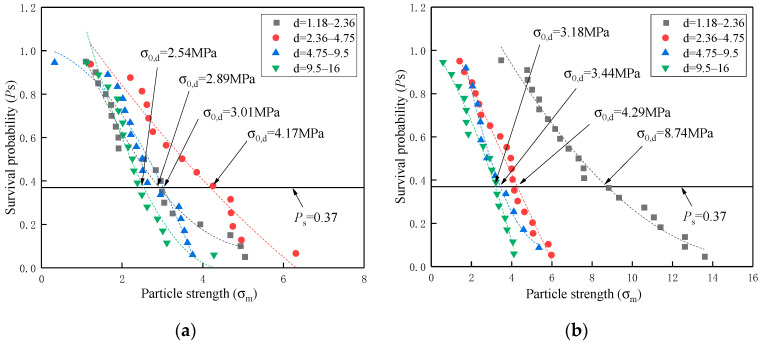
The curves of survival probability to crushing strength of coral parent rock particles: (**a**) without wrapping cement slurry; (**b**) with wrapping cement slurry.

Corresponding to the crushing strength $\sigma_{f}$, Equation (9) demonstrated that the survival probability $P_{s}$ was assigned as a cumulative value of the *i*th result in the set of n samples. As a further explanation, $P_{s}$ was defined as the proportion of crushed elements to the whole single particle. Figure 8 and Figure 9 show the $P_{s}-\sigma_{f}$ curves of different coral particles with and without cement slurry wrapped in various size ranges, respectively. Meanwhile, the characteristic crushing strength $\sigma_{0,\;d}$ was highlighted as points of intersection between line $P_{s}=37\%$ and curves.

There is a general trend that the characteristic crushing strength $\sigma_{0,\;d}$ was in inverse correlation with particle size. Such inverse correlation is one typical strength characteristic of brittle material and is consistent with the previous research on coral sand from the South China Sea [15,16]. Meanwhile, almost all data supported another empirical law [28,32] that fine particles demonstrated a higher survival probability $P_{s}$ than coarse particles did at a certain characteristic crushing strength $\sigma_{0,d}$, regardless of the tested groups and particle categories. However, there is only one exceptional data set in which the parent rock particles (1.18–2.36 mm) started with a relatively lower $\sigma_{0,\;d}$ value of 2.89 MPa, resulting in partial inconformity with the aforementioned empirical laws. One reasonable explanation was that such size particles were attributed to the corrosive exfoliation from the larger parent rock surface, therefore characterized with lower original crushing strength and more brittleness. In addition, the parent rock particles have significant structural properties, and the mechanical properties of the parent rock particles are significantly diminished after disturbance [8].

With further data analysis, the results of $\sigma_{0,d}$ in Table 8 revealed several physical phenomena for profoundly describing the hardening mechanism of coral single particle with wrapping cement slurry. Firstly, the wrapped cement slurry universally demonstrated the enhancement of characteristic crushing strength $\sigma_{0,d}$ regardless of tested groups and particle categories. Secondly, both wrapped single particles of coral biological debris and coral parent rock achieved extremely high $\sigma_{0,d}$ values of 10.14MPa and 8.74 MPa, respectively, in the size range from 1.18 mm to 2.36 mm. On the one hand, such high $\sigma_{0,d}$ values were twice or three times more than any other values of 2.36–16 mm. On the other hand, the wrapped parent rock particles (1.18 mm–2.36 mm) presented a much higher enhancement ratio of $\sigma_{0,d}$ than debris particles (1.18–2.36 mm). As an explanation, the fine particles of 1.18–2.36 mm meant more abundant surface open pores to allow fuller growth of calcium hydroxide crystals. Furthermore, the parent rock particles (1.18–2.36 mm) showed low original crushing strength. Thirdly, with the increased particle size, the wrapped debris particles tended to display a higher value of $\sigma_{0,d}$ and enhancement ratio than the wrapped parent rock particles. The reason for this phenomenon could be also considered as the increased disparity of specific surface area and pore volume between natural debris particles and parent rock particles with the increased particle size.

Furthermore, the crushing strength of coral particles in previous studies and this work can be seen in Table 9. The coral biological debris chosen for this work has a lower particle crushing strength than calcareous coral particles from other regions. Besides, as the particle size increases, the crushing strength decreases noticeably, which is similar to other studies.

#### 3.3.3. Weibull Statistical Analysis

**Figure 10:**
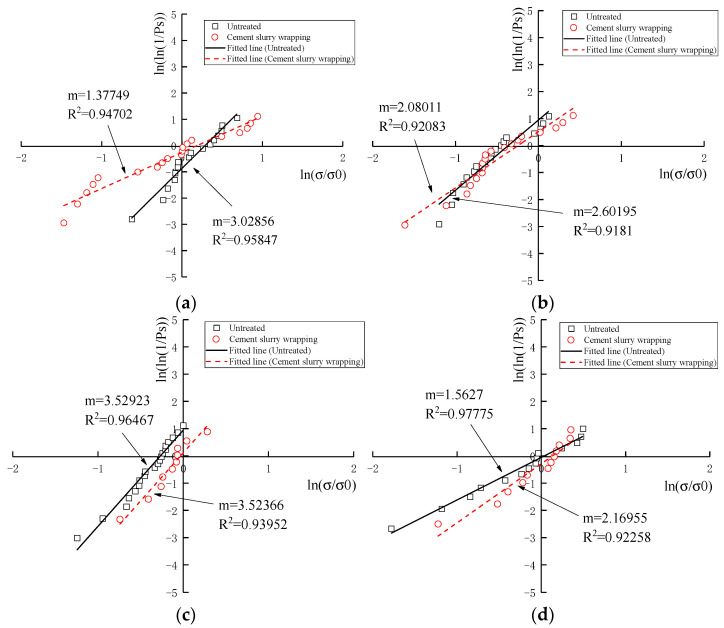
Weibull distribution linearity of normalized survival probability to normalized particle crushing strength of coral biological debris particles: (**a**) 1.18–2.36 mm; (**b**) 2.36–4.75 mm; (**c**) 4.75–9.5 mm; (**d**) 9.5–16 mm.

**Figure 11:**
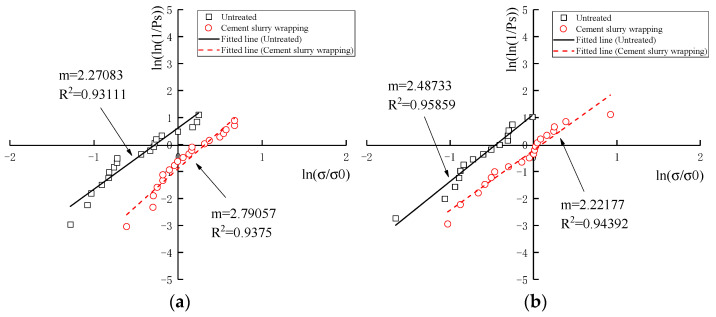

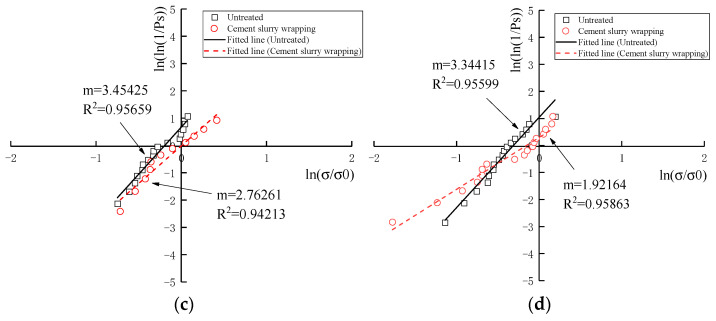
Weibull distribution linearity of normalized survival probability to normalized particle crushing strength of coral parent rock particles: (**a**) 1.18–2.36 mm; (**b**) 2.36–4.75 mm; (**c**) 4.75–9.5 mm; (**d**) 9.5–16 mm.

To accomplish Weibull statistical analysis, the abovementioned measured crushing strength $\sigma_{f}$ and survival probability $P_{s}$ were included in the empirical correlation (Equation (7)) of the Weibull breakage distribution under tensile stress to calculate the Weibull modulus m. It is a significant index to describe the inverse proportion with strength variability. Equation (7) includes double logarithms on each side and was subsequently simplified as Equation (10). Correspondingly, the $P_{s}-\sigma_{f}$ curves in Figure 10 and Figure 11 were normalized into a linear regression of $\ln\left[\ln\left(1/P_{s}\right)\right]$-$\ln\left(\sigma_{f}/\sigma_{0}\right)$, directly presenting the Weibull modulus m and the correlation index $R^{2}$. No matter which tested groups and particle categories are considered, all linear regression models demonstrated a positive correlation with similar trajectories, an excellent Weibull modulus m and a high correlation index $R^{2}$. The Weibull modulus m ranging from 1.38 to 3.45 was extremely similar to those m values of previous studies on coral sand from South China Sea [15,16]. Meanwhile, the correlation indexes $R^{2}$ are distributed from 0.9181 to 0.9778, representing the reliable linearity of Weibull regression models.

(10) $$\ln\left[\ln\left(\frac{1}{P_{s}}\right)\right]=m*\ln\left(\frac{\sigma }{\sigma_{0}}\right)$$

With further data analysis, the results of m, $\sigma_{m}$ in Table 10 presented several somewhat unexpected but thought-provoking phenomena for the establishment of Weibull statistical models on a brittle single coral particle.

Firstly, no matter which tested groups and particle categories are considered, the wrapped cement slurry commonly reduced the Weibull Modulus m, but increased the strength variability. However, the Weibull modulus *m* showed an increase for coral debris particles with particle sizes of 9.5–16 mm, and for parent rock particles with particle sizes of 1.18–2.36 mm. For the coral debris particles (9.5–16 mm), the dispersion of crushing strength was quite high. However, the shape was relatively regular after being wrapped cement slurry, making the dispersion relatively modest. For the coral parent rock (1.18–2.36 mm), the crushing strength was greatly increased after wrapping cement slurry, and the crushing strength was mainly shown as the strength of cement slurry, which has less dispersion.

Secondly, the Weibull modulus m was weakly correlative to particle size range regardless of tested groups and particle categories. This conclusion was in disagreement with most previous research of coral sand. For example, Zhang [28] stated that the Weibull modulus m was directly proportional to particle size; by contrast, Ma [16] insisted on their inverse proportion. However, Wu [15] presented similar data sets with a weak relationship between the m value and the particle size. In our opinion, more definite conditions should be proposed before discussing their relationship, as an alternative, additional critical relevant parameter could be introduced as a bridge between them.

Thirdly, the Weibull normalized effective crushing strength $\sigma_{m}$ of a coral single particle demonstrated similar values and consistent tendencies of characteristic crushing strength $\sigma_{0,d}$. Therefore, it is of great convenience to regard $\sigma_{0,d}$ values when $P_{s}=37\%$ as a quick-inspection index to estimate $\sigma_{0,d}$ values in Weibull statistical analysis.

### 3.4. Preparation and Properties of Coral Concrete

In terms of coral concrete preparation, there has been fundamental agreement that the special characteristics, such as large specific surface area and abundant surface porosity, would impose an extra cement dosage of about 30–40% when ensuring the same strength grade of coral concrete as conventional cement concrete [4]. On this basis, the object of subsequent experiments was to explore the variation in compressive strength and destroy the form of coral concrete with increasing cement dosage. As compiled in Table 11, the main construction ingredients comprised water, cement, coral debris particles, pre-wetting coral parent rock particles and a water-reducing admixture. Technically, cement dosage was applied by more than 500 kg/m^3^ to obtain the strength grade C30 of coral concrete similar to conventional cement concrete. Last but not least, the particle proportion of coral debris to parent rock was ensured as high as 3:2 to promote the particle grade of coral aggregates (d < 19 mm), increase concrete mobility during mixing and strengthen the compressive strength of coral concrete.

**Figure 12:**
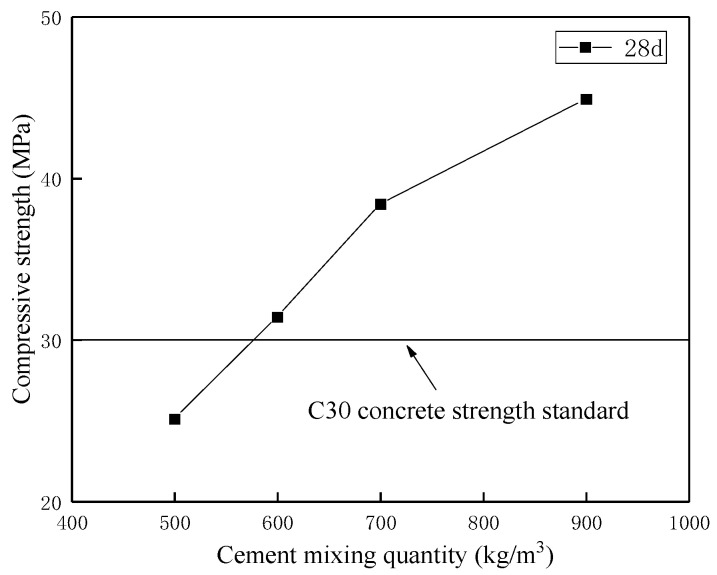
The variation in compressive strength with cement dosage.

Another noteworthy point was that the parent rock particle was pre-wetted to satisfy its outstanding water absorbency. On the one hand, it is more convenient to determine the consistent water usage for both categories of coral aggregates during mixing. On the other hand, more water volume was prepared for supporting secondary hydration reactions and increasing the long-term concrete compressive strength. As illustrated in Figure 12, the compressive strength of coral concrete was initially in direct proportion to cement dosage; subsequently, the gradient of strength to dosage tended to decrease when cement dosage was more than 700 kg/m^3^. Likewise, the strength mechanism of coral concrete is similar to that of conventional cement concrete, whose compressive strength decreased with increasing the water-cement ratio.

**Figure 13:**
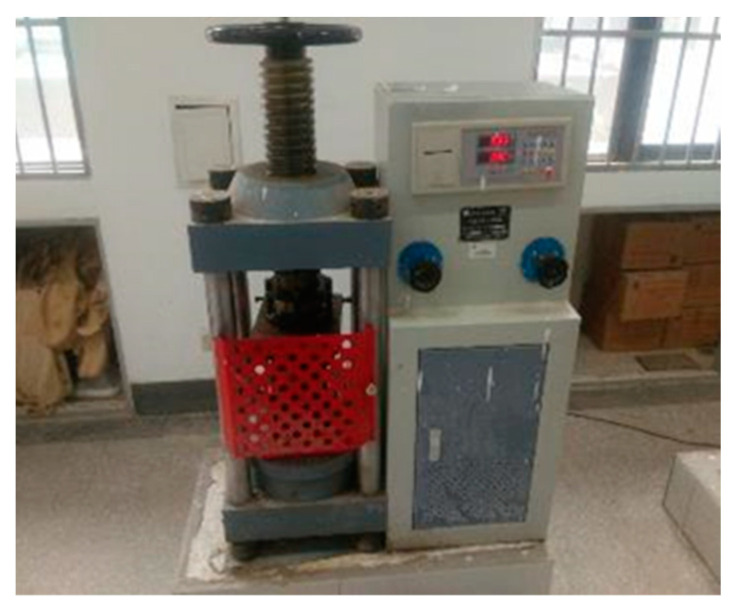
The instruments of the compressive strength test.

**Figure 14:**
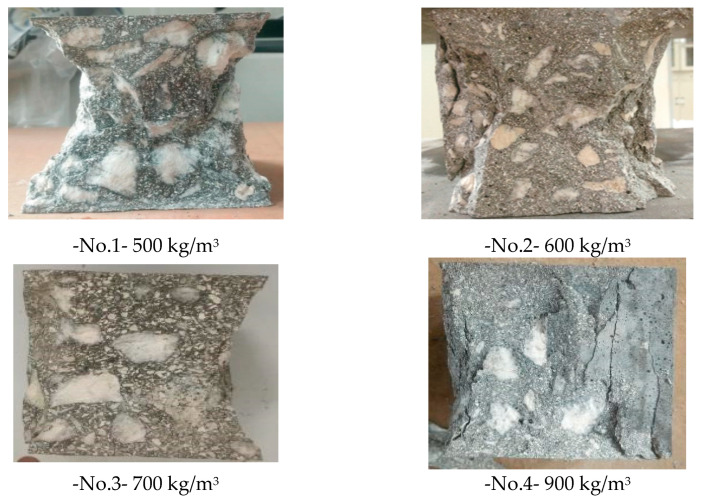
Failure surfaces of coral concrete.

The instruments can be seen in Figure 13. Moreover, Figure 14 was to compare the failure mechanism among four specimens of coral concrete with varied cement dosages. As opposed to conventional cement concrete, the failure surface of coral concrete arose directly through aggregates rather than along their profiles, which may be a result of the relatively low crushing strength of coral aggregates. Although this phenomenon meant the weakness of the coral concrete skeleton, the compressive strength of most specimens still achieved the requirement of standard strength grade C30. Therefore, the hardening mechanism of coral aggregates took over this responsibility for principal structural support in coral concrete. Another noteworthy phenomenon was the several cracking of coarse coral aggregates and shear failure in fine ones, which was equivalent to the performance of ordinary concrete with a low strength grade and gravel aggregates. However, there was a similar phenomenon between coral concrete and conventional cement concrete. Figure 14 illustrates a tendency towards a smaller angle of inclination of failure surfaces with increasing cement dosage and decreasing water-cement ratio. Referring to Figure 12, the explanation could be that the appropriate proportion of particles ensured concrete mobility during mixing; meanwhile, the increased cement dosage allowed for more hydration reactions, a full hardening process and higher compressive strength.

### 3.5. Interfacial Transition Zone (ITZ) of Coral Concrete

Coral aggregate, which generally includes coral parent rock particles, is a typical soft material with a low hardness and shear strength; hence, it fails to support the principal structure as a concrete skeleton as conventional cement concrete does. In turn, coral aggregates cause the “substrate effect” and “micro-pump” mechanism to concrete due to abundant open pores and rough surfaces. On the one hand, the substrate formed by such open pores and surfaces allows more space for the cross-densification effect of hydration crystals in ITZs. On the other hand, the coral concrete is granted a property of distinct water absorbency in ITZs, which induces the partial appearance of micro-pumps at the early and final stage of hydration reactions [34]. In comparison to conventional cement concrete, the micro-pump mechanism is of great benefit to promote water absorption, avoid the formation of water films, reduce the partial water–cement ratios and densify hydration crystals in ITZs at the early stage of hydration reactions [35]. Meanwhile, such a mechanism can provide much water conserved during primary hydration reactions to support secondary hydration reactions in ITZs under an enriched-slurry concrete system. Therefore, both the micro-pump mechanism and the substrate effect of coral aggregates should be taken into account when analysing the formation of ITZs’ strength. More significantly, there is a great need to establish a different conceptual model of coral concrete due to such a special crystal gain mechanism in ITZs during hydration reactions as well as the soft material characteristics of low strength and hardness. The coral concrete prepared for the nanoindentation tests was composed of PII42.5R cement with 1%, pre-wetting coarse particles of coral parent rock (4.75 mm ≤ d < 19 mm) with 0.82%, fine particles of coral biological debris (d < 4.75 mm) with 1.23%, and the water-reducing agent with 0.05%.

**Figure 15:**
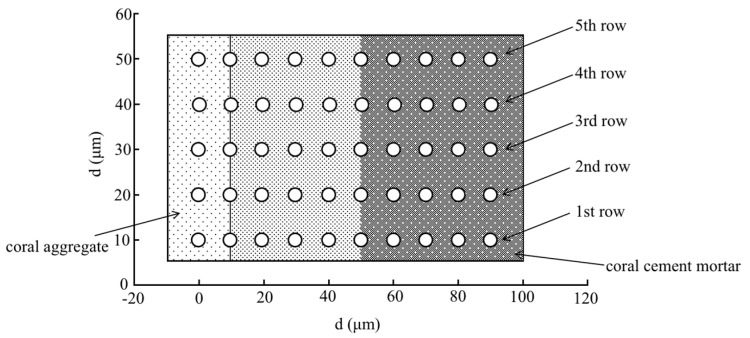
Schematic diagram of nanoindentation test points.

Building on sub-microscopic research on this crystal gain mechanism in ITZs during hydration reactions, this study implemented a nanoindentation test to reveal the influence of the encounter of coral particles and alkaline cement material on coral concrete characteristics. Figure 15 is to demonstrate the distribution of test points of coral aggregate (left) and coral cement mortar (right). The test points were arranged with an interval of 10μm both horizontally and vertically. The test point was skipped when encountered with unreacted cement particles. The data points of five rows and nineteen columns were collected.

**Figure 16:**
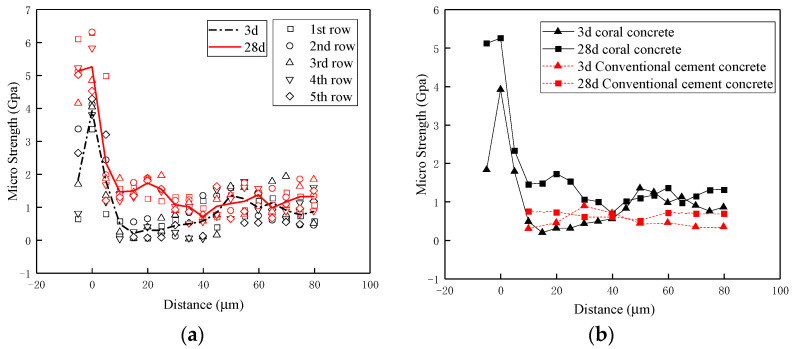
Schematic diagram of nanoindentation test points: (**a**) nanoindentation results with different curing ages; (**b**) comparison between coral concrete and ordinary concrete.

Figure 16a is simultaneously presented for the data results of nanoindentation tests of coral concrete specimens with different maintenance ages of 3 and 28 days. Technically, the origin of the horizontal coordinate (d = 0) is the intermediate interface of coral aggregates and the ranges d = −10–10 μm, d = 10–50 μm, d > 50 μm, respectively, represent coral aggregate zone, ITZs and coral cement mortar zone.

As is illustrated in Figure 16a, the coral concrete specimens with 3-day maintenance displayed a relatively larger variability of sub-microscopic strength. Since 3-day maintenance meant the early stage of hydration reactions, ITZs (d = 10–50 μm) were still classified into weak areas with lower strength around 0.5 GPa compared to that of coral cement mortar zone (d > 50 μm) around 1 GPa. On the contrary, the sub-microscopic strength of the coral aggregate zone (d = −10–10 μm) reached a significantly high level around 2 GPa, particularly that of intermediate interface of coral aggregates (d = 0 μm) up to 3.3–4.3 GPa. On the other hand, in terms of the coral concrete specimens with 28-day maintenance, ITZs (d = 10–50 μm) demonstrate a relatively narrower fluctuation range of sub-microscopic strength and a well-enhanced value of more than 1 GPa due to the near accomplishment of hydration reactions. In the meantime, the sub-microscopic strength of the coral aggregate zone (d = −10–10 μm) climbed up to 5–6 GPa. Furthermore, it can be seen in Figure 16b that the strength of the ITZs of coral concrete was marginally higher than that of conventional concrete at the curing age of 28 days. Besides, the coral concrete’s strength in the cement mortar area was greater than that of conventional concrete at the curing ages of 3 and 28 days.

In summary, the specific gravity of coral particles is comparable to that of quartz sand, and there are not many differences between coral particles’ specific gravities at different particle sizes. However, for the specific surface area and water absorption rate, coral particles differ greatly from common quartz sand. Additionally, the specific surface area and open pores of coral debris particles are bigger than those of coral parent rock particles. The specific surface area and open pores of coral particles increase when particle sizes are reduced. Therefore, to fill the open pores of the particles and encapsulate them, it is necessary to increase the cement admixture in the concrete system when producing coral concrete, if the proportion of fine particles rises. Because of the higher water absorption rate of coral particles, the water content of the concrete system must be increased.

The conceptual model of conventional cement concrete is widely used as an experimental basis in research. It is composed of coarse aggregate, filled cement mortar and an interfacial transition zone (ITZ) [20]. In general, the coarse aggregate plays a vital role as concrete a skeleton for principal structural support and the filled cement mortar is for internal connections. Additionally, the strength of coral particles was improved after wrapping cement slurry, especially for the coral fine particles. However, for the coral coarse particles, the enhancing effect is not obvious and the strength of coral coarse particles is modest. Therefore, the coarse particles are unable to serve as skeleton support in coral concrete. The strength of the concrete is mostly provided by coral fine particles and cement mortar. Therefore, an increase in the content of coral fine particles can lead to a greater strength of coral concrete.

**Figure 17:**
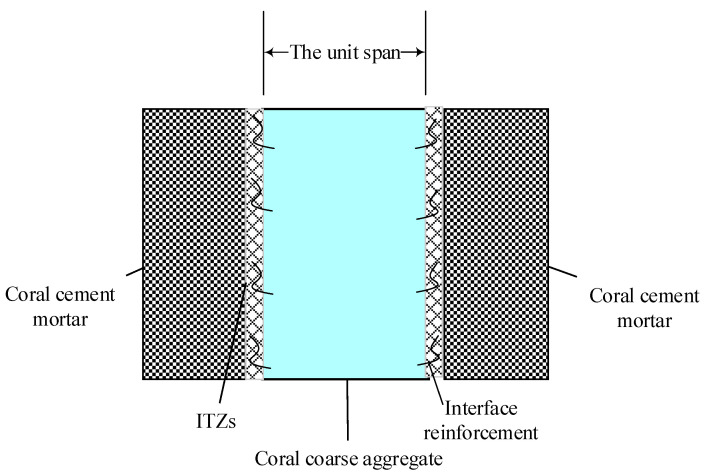
The coral concrete frame and structure model.

According to the above analysis, the strength of coarse particles in coral concrete systems is less than that of conventional concrete, and even less than that of coral cement mortar. Consequently, the conceptual model of coral concrete is different from that of conventional concrete. The ternary structure of the coral concrete system is defined in accordance with the aforementioned characteristics of soft and low strength of coral coarse aggregate, the strengthening mechanism of the ITZs of cement. As can be seen in Figure 17, the structural model contains coral coarse aggregate, coral cement mortar, and ITZs.

Firstly, in the rich cement mortar coral concrete system, the coral cement mortar replaces the coral aggregate as the framework support unit because it has much higher strength than the soft and low-strength coral aggregate. Additionally, the size of the coral coarse aggregate determines the unit span of the coral cement mortar framework of the coral concrete system. Secondly, because of the multiple improvement mechanisms of the “substrate effect” on the surface of coral aggregate, the formation, adhesion and growth of the hydration products were promoted. The hydration products in the ITZs of coral aggregate are no longer the weak zone of the coral concrete system because of their compact structure and reliable cementation.

## 4. Conclusions

To study the hardening mechanism of coral particles in the cement-based system, in this study, a series of elementary physical experiments were implemented to compare the properties of two different types of coral particles. Additionally, this study used particle crushing strength tests, compressive strength tests, and nanoindentation tests to investigate the hardening mechanism of coral particles. Eventually, the conclusions were summarized as follows:

(1) The mineral elements of coral particles were primarily aragonite and high-magnesian calcite, with an equivalent carbonate concentration exceeding 97%. Both debris and parent rock particles had a larger specific surface area and open pore volume. Additionally, the debris particles generally had higher surface porosity and irregular geometry. However, the water absorption rate of debris particles was 9.9%, which was lower than that of parent rock particles.

(2) The crushing strength of coral particles after wrapping with cement slurry was higher than that in the untreated state, but the crushing strength of parent rock particles is significantly lower than that of debris particles. Furthermore, because of the fine coral debris particles (1.18–2.36 mm) with higher self-strength, the particle crushing strength *σ*_0,*d*_ reached 9.11 MPa, and after wrapping with cement slurry, the crushing strength *σ*_0,*d*_ reached 10.04 MPa. For the fine parent rock particles (1.18–2.36 mm), the *σ*_0,*d*_ before wrapping with cement slurry was only 2.89 MPa, but the strength of the wrapped cement slurry was significantly enhanced, reaching 8.74 MPa. However, the strength-enhancing effect was not obvious for the coral particles (>2.36 mm).

(3) The appropriate proportion of coral particles ensured concrete mobility during mixing. Meanwhile, the increased cement dose enabled coral concrete to harden completely and have a higher compressive strength. Due to the low strength of the coral coarse aggregate, it cannot serve as a skeletal support. In other words, the hardening mechanism of coral aggregates, especially fine particles and coral cement mortar, took over the responsibility for structural support in coral concrete.

(4) Nanoindentation tests revealed the great enhancement of sub-microscopic strength in ITZs. The “micro-pump” effect on the surface of coral aggregate contributes to the formation of the ITZs in coral concrete. The ITZs of coral aggregate are still low-strength and weak in the early stages of the hydration reaction, but with the increase in maintaining age, the strength increases substantially.

## Figures and Tables

**Table 1:** The particle size distribution of coral particles.

**Sieve Diameter (mm)**	19.0	16.0	9.5	4.75	2.36	1.18	0.6	0.3	0.15	0.075
**Passing Percentage (%)**	100	84.8	74.8	58.9	49.5	38.9	33.6	15.3	6.5	1.8

**Table 2:** The physical properties of cement.

Test Content	Specific Surface Area (kg/m^2^)	Loss on Ignition (%)	Setting Time (min)		Compressive Strength (MPa)		Flexural Strength (MPa)	
			Initial	Final	3d	28d	3d	28d
Standard	≥300	≤5.0	≥45	≤390	≥22	≥42.5	≥4.0	≥6.5
Result	394	3.0	140	195	33.7	53.6	6.5	9.1

**Table 3:** The basic properties of Sika ViscoCrete 540P.

Appearance	Bulk Density (kg/m^3^)	pH Value	Cl^−1^ Contents	NaCl Contents	Water Reducing Rate
White powder	600	10.5	0.1%	2.0%	30%

**Table 4:** The variation of specific surface area and pore volume with particle size ranges.

Particle Size Range (mm)	Specific Surface Area (m^2^/kg)		Pore Volume (cm^3^/kg)	
	Debris	Rock	Debris	Rock
<0.075	1867.6	/	12.186	/
0.075–0.15	1114.3	/	7.085	/
0.15–0.3	1159.3	/	4.986	/
0.3–0.6	536.0	/	2.660	/
0.6–1.18	481.9	169.0	3.829	1.262
1.18–2.36	458.2	129.1	3.205	0.929
2.36–4.75	470.1	132.6	3.483	0.742
4.75–9.5	497.0	88.0	2.558	0.250
Standard sand (<2.36)	189.0		0.634	

**Table 5:** Specific gravity of coral particles.

Boundary Particle Size (mm)		Debris Specific Gravity	Rock Specific Gravity
Fine grain Group S	0.075–0.6	2.98	/
	0.6–2.36	2.97	2.45
	2.36–4.75	2.94	2.20
Coarse grain Group G	4.75–9.5	2.75	2.19
	9.5–16	2.60	2.10

**Table 6:** Water absorption rate of coral particles.

Particle Size (mm)	Water Absorption Rate (%)	
	Debris Particles	Rock Particles
2.36–4.75	8.69	24.04
4.75–9.5	9.50	24.56
9.5–16	11.50	17.46
Average (%)	9.90	22.02

**Table 7:** The mass proportion of various chemical compounds in coral particles.

Compounds	CaO	MgO	SrO	SO_3_	Na_2_O	SiO_2_	Al_2_O_3_
Proportion (%)	47.1	2.48	0.63	0.47	0.30	0.13	0.060
Compounds	P_2_O_5_	Cl	K_2_O	Fe_2_O_3_	CO_2_(Estimated amount of burn-off)		
Proportion (%)	0.060	0.026	0.0082	0.030	48.7		

**Table 8:** Relevant parameter *σ*_0,*d*_ of crushing strength of coral particles.

Particle Size Range (mm)	Wrapped Cement Slurry	*σ*_0,*d*_ (MPa)	
		Biological Debris	Parent Rock
1.18–2.36	Before	9.11	2.89
	After	10.04	8.74
2.36–4.75	Before	4.35	4.17
	After	6.25	4.29
4.75–9.5	Before	2.57	2.89
	After	3.93	3.44
9.5–16	Before	3.61	2.54
	After	4.75	3.18

**Table 9:** The statistics of crushing strength of coral particles.

Particle Type	Particle Size (mm)	Average Particle Size (mm)	$\mathrm{Particle}\;\mathrm{Crushing}\;\mathrm{Strength}\;\sigma_{0,d}$ (MPa)	Region	Reference
Calcareous coral particles	5–8.45 mm	6.7 mm	6.54	South China Sea	[15]
	8.45–10.5 mm	9.5 mm	6.26		
	10.5–14.5 mm	12.5 mm	5.12		
Calcareous coral particles	3–5 mm	4 mm	5.67	South China Sea	[16]
	6.5–8.5 mm	7.5 mm	4.86		
	10–12 mm	11 mm	4.34		
Calcareous coral particles	1.53–1.95 mm	1.7 mm	11.23	South China Sea	[33]
	2.94–4.42 mm	3.7 mm	7.31		
	7.90–9.97 mm	8.9 mm	4.62		
Coral biological debris	1.18–2.36 mm	1.7 mm	9.11	South China Sea	This work
	2.36–4.75 mm	3.5 mm	4.35		
	4.75–9.5 mm	7.1 mm	2.57		
	9.5–16 mm	12.7 mm	3.61		

**Table 10:** Relevant parameters m, σm of crushing strength of coral particles.

Particle Size Range (mm)	Wrapped Cement Slurry	Biological Debris		Parent Rock	
		m	σ_m_/MPa	m	σ_m_/MPa
1.18–2.36	Before	3.03	8.14	2.27	2.56
	After	1.38	9.17	2.79	7.78
2.36–4.75	Before	2.60	3.86	2.49	3.70
	After	2.08	5.54	2.22	3.80
4.75–9.5	Before	3.53	2.31	3.45	2.60
	After	3.52	3.54	2.76	3.06
9.5–16	Before	1.56	3.24	3.34	2.28
	After	2.17	4.21	1.92	2.82

**Table 11:** The ingredient proportion of coral concrete preparation.

No.	Cement (kg/m^3^)	Coral Biological Debris Particles (kg/m^3^)	Coral Parent Rock Particles (kg/m^3^)	Water-Cement Ratio	Water-Reducing Admixture (%)
1	500	729	486	0.4	0.05
2	600	686	457	0.35	0.05
3	700	643	429	0.3	0.05
4	900	600	400	0.25	0.05

## Data Availability

Not applicable.

## References

[1] Wang X.C. (2008). Engineering Geological Characteristics of Coral Reefs in Nansha Islands and Feasibility Study of Large-Scale Engineering Construction. Ph.D. Thesis.

[2] Li L. (2012). Study on the Basic Properties of Coral Concrete. Ph.D. Thesis.

[3] Sun Z. (2000). Engineering properties of coral sands in Nansha Islands. Trop. Ocean.

[4] Liu J., Ou Z., Peng W., Guo T., Deng W., Chen Y. (2018). Literature Review of Coral Concrete. Arab. J. Sci. Eng..

[5] Wang L., Zhao Y., Lv H. (2012). Basic properties and research application prospects of coral aggregate concrete. Concrete.

[6] Todisco M.C., Wang W., Coop M.R., Senetakis K. (2017). Multiple contact compression tests on sand particles. Soils Found..

[7] Zhou B., Ku Q., Wang H., Wang J. (2020). Particle classification and intra-particle pore structure of carbonate sands. Eng. Geol..

[8] Xiao X., Zhang R., Peng D. (2018). Research on Geotechnical Characteristics of Maldives Coral Reef. Railw. Surv..

[9] Jin Y., Chen T., Meng Q., Hu M. (2017). Uniaxial compressive strength reveals the differences of coral skeleton structure in the South China Sea. J. Trop. Oceanogr..

[10] Ren S., Xi W., Cao Z. (2016). Analysis of Physical and Mechanical Properties of Coral Reef and Engineering Application. J. Changchun Inst. Eng. (Nat. Sci. Ed.).

[11] Wang Y., Hong Y., Guo Z., Wang L. (2018). Macro and meso fracture mechanical properties of South China Sea calcareous sand. Geotech. Mech..

[12] Jiang M.J., Wu D., Cap P., Ding Z.J. (2017). Analysis of connected pores of calcareous sand based on SEM images. J. Geotech. Eng..

[13] Cao P., Ding Z. (2019). Research on pore distribution characteristics of calcareous sand based on MIP and CT tests. J. Water Resour. Build. Eng..

[14] Chen H., Wang R., Li J., Zhang J. (2005). Shape analysis of calcareous sand particles. Geotech. Mech..

[15] Wu X., Cai Y., Xu S., Zhuang Y., Wang Q., Wang Z. (2021). Effects of size and shape on the crushing strength of coral sand particles under diametral compression test. Bull. Eng. Geol. Environ..

[16] Ma L., Li Z., Wang M., Wei H., Fan P. (2019). Effects of size and loading rate on the mechanical properties of single coral particles. Powder Technol..

[17] Li Y., Li S., Liu X., Chen W. (2020). Experimental Study on the Effect of Particle Crushing on the Compressive Properties of Calcareous Sand. J. Eng. Geol..

[18] Li S., Liu F., Dai X., Zhang Y. (2019). Laboratory experimental study on the influence of calcium carbonate content on the properties of calcareous sand. Exp. Mech..

[19] Yu D., Yi J., Song Z., Shen N., Zhang J., An B. (2020). Effect of aggregate particle size on mechanical properties of coral concrete. J. East China Jiaotong Univ..

[20] Wang L., Cao M. (2006). Research on Concrete Structure Model. J. Wuhan Univ. Technol..

[21] Wang L., Mei J., Wu J., He X., Li H., Ding Q. (2020). Mechanical Properties and Microscopic Mechanism of Coral Sand-Cement Mortar. Adv. Mater. Sci. Eng..

[22] Zhang X., Liu X., Xu Y., Wang G., Zang M. (2022). Fragmentation modes of single coral particles under uniaxial compression: Microstructural insights. Constr. Build. Mater..

[23] Gao Y., Shi T., Li W., Chen Q. (2022). Mesoscopic characteristics of particle breakage in calcareous sand during compression. J. Hydroelectr. Power.

[24] (2006). Standard for Technical Requirements and Test Method of Sand and Crushed Stone (or Gravel) for Ordinary Concrete.

[25] (2017). Determination of the Specific Surface Area of Solids by Gas Adsorption Using the BET Method.

[26] Chen J., Zhang J. (2009). Application of ASAP2020 Specific Surface Area and Pore Analyzer. Anal. Instrum..

[27] (2019). Standard for Soil Test Methods.

[28] Zhang J. (2004). Research on the Basic Mechanical Properties of Calcareous Sand and the Impact of Particle Breakage. Ph.D. Thesis.

[29] Weibull W. (1951). A Statistical Distribution Function of Wide Applicability. Appl. Mech..

[30] Hu X., Li L., Yang S. Weibull-strength size effect and common problems with size effect models. Proceedings of the International Conference on Fracture Mechanics of Concrete & Concrete Structures.

[31] Aliha MR M., Sistaninia M., Smith D.J., Pavier M.J., Ayatollahi M.R. (2012). Geometry effects and statistical analysis of mode I fracture in guiting limestone. Int. J. Rock Mech. Min. Sci..

[32] Nakata Y., Hyde A., Hyodo M. (1999). A probabilistic approach to sand particle crushing in the triaxial test. Geotechnique.

[33] Chen H., Wei H., Feng Z., Zou Y. (2018). Study on crushing strength and crushing form of calcareous sand particles. Geotech. Found..

[34] Vandamme M., Ulm F.-J., Fonollosa P. (2010). Nanogranular packing of C–S–H at substochiometric conditions. Cem. Concr. Res..

[35] Li L., Zhao Y., Lu H., Han C. (2011). Effect of coral aggregate pre wetting on mechanical properties of concrete. Concrete.

